# Shiftwork and Light at Night Negatively Impact Molecular and Endocrine Timekeeping in the Female Reproductive Axis in Humans and Rodents

**DOI:** 10.3390/ijms22010324

**Published:** 2020-12-30

**Authors:** Alexandra M. Yaw, Autumn K. McLane-Svoboda, Hanne M. Hoffmann

**Affiliations:** Department of Animal Science and the Reproductive and Developmental Science Program, Michigan State University, East Lansing, MI 48824, USA

**Keywords:** fertility, women, rodent, shiftwork, estrous cycles, circadian disruption, infertility, menstrual cycles, pregnancy, birth

## Abstract

Shiftwork, including work that takes place at night (nightshift) and/or rotates between day and nightshifts, plays an important role in our society, but is associated with decreased health, including reproductive dysfunction. One key factor in shiftwork, exposure to light at night, has been identified as a likely contributor to the underlying health risks associated with shiftwork. Light at night disrupts the behavioral and molecular circadian timekeeping system, which is important for coordinated timing of physiological processes, causing mistimed hormone release and impaired physiological functions. This review focuses on the impact of shiftwork on reproductive function and pregnancy in women and laboratory rodents and potential underlying molecular mechanisms. We summarize the negative impact of shiftwork on female fertility and compare these findings to studies in rodent models of light shifts. Light-shift rodent models recapitulate several aspects of reproductive dysfunction found in shift workers, and their comparison with human studies can enable a deeper understanding of physiological and hormonal responses to light shifts and the underlying molecular mechanisms that may lead to reproductive disruption in human shift workers. The contributions of human and rodent studies are essential to identify the origins of impaired fertility in women employed in shiftwork.

## 1. Introduction

Millions of Americans are currently employed in nighttime (nightshift) or rotating shiftwork, a number which continues to rise [1], increasing our need to understand links between the increased risk for numerous health disorders associated with shiftwork, such as reduced fertility. For decades, the clinical implications of shiftwork on reproductive dysfunction have been examined. Most of this work has focused on the female reproductive system, although some reports also show an impact on male reproductive status [2,3]; however, this literature is less explored. While several studies have provided strong correlational relationships between female shift workers and reproductive deficits throughout the reproductive cycle, including menstrual/ovulatory cycling [4,5,6,7,8,9], fertility and pregnancy [10,11,12,13], as well as labor and birth [14,15,16,17,18], there are still significant gaps in our knowledge of the mechanisms by which shiftwork may cause reduced reproductive function. Light at night or frequent changes in light cycles (i.e., shiftwork) are hypothesized to cause a repeated mismatch between the cellular circadian rhythms and the daily timing signals received from the master circadian pacemaker in the brain, the suprachiasmatic nucleus (SCN). These repeated mistimed light signals disrupt the synchrony of rhythms in the body and leads to impairments and mistiming of physiological functions. Although clinical data from shift workers have documented the influence of shiftwork on several aspects of reproductive function and fertility, animal models are necessary to piece apart the mechanistic and potential causal role of shiftwork on reproductive dysfunction. In this review, we aim to provide a summary of the impact of shiftwork on female fertility. An in-depth analysis of clinical data is compared to rodent models of shiftwork to discuss insights and challenges with current rodent models of shiftwork and their ability to recapitulate shiftwork in humans.

### 1.1. Light and Molecular Clocks

**Figure 1:**
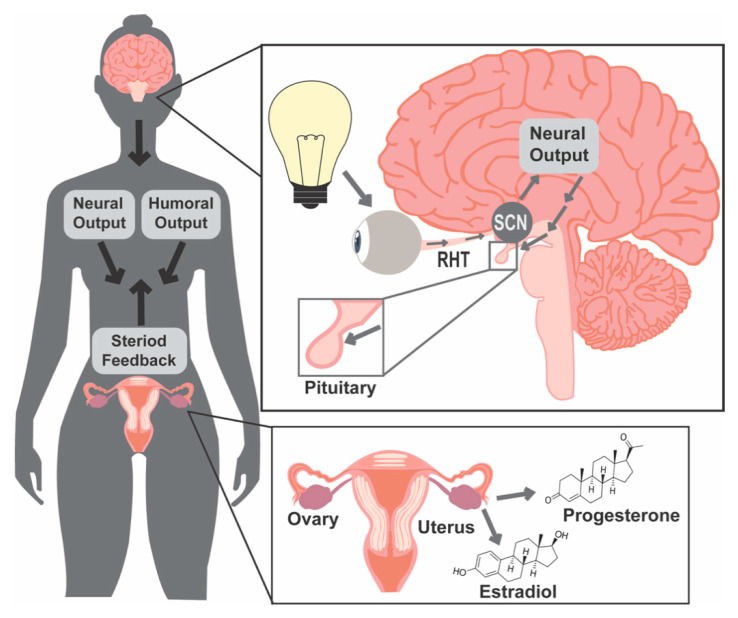
Photic information regulates the female reproductive system. Light enters the eye, where it is captured by the non-vision, intrinsically photosensitive retinal-ganglion cells (ipRGCs) of the retina. Photic information is transmitted directly from ipRGC’s, through the retinohypothalamic tract (RHT), to the brain, including the suprachiasmatic nucleus (SCN) of the hypothalamus. The SCN translates light information and sends signals to non-SCN brain areas and peripheral tissues via neuronal and humoral pathways, allowing target tissues to entrain to time of day. In turn, reproductive tissues, including the ovary and uterus, respond to such cues. The ovary provides steroid hormone feedback to this axis through estrogens and progesterone, which can modulate behavior as well as the timing of central and peripheral clock function.

Two main aspects of shiftwork, rotating between day/night schedules and exposure to light at night, are thought to be the origins of health deficits in shift workers, which include increased risk of numerous endocrine disorders, such as diabetes [19,20], cardiovascular disease [21,22,23], breast cancer [24,25,26,27] and reduced fertility [10]. Most physiological functions are governed by biological rhythms, which enable organisms to fine-tune their behavioral, physiological, and cellular processes to the external environment, including ultradian (less than a day), circadian (daily), and infradian (longer than a day, such as annual or seasonal) rhythms. Evolutionary adaptation of plants and animals to the Earth’s 24 h day/night rotation has resulted in the development of circadian timekeeping systems. One of the strengths of these systems is their high sensitivity to photic cues, which encode information based on light timing, intensity, and duration. To synchronize physiological functions to the time of day, light information is captured by the eye and transmitted through the retinohypothalamic tract to the SCN. The SCN transmits daily timing signals to non-SCN brain areas and peripheral tissues to allow for the coordination of events occurring throughout the body, including hormone release and timed function of cells and tissues within the female reproductive system (Figure 1). Unfortunately, in today’s industrialized world, the high sensitivity of the circadian timekeeping system to light has turned into a modern-day weakness of this system. Light is no longer restricted to the daytime and most animals and humans experience extensive exposure to artificial light at night. This increase in nocturnal, mistimed photic cues impacts SCN timekeeping function, causing a mismatch between the expected environmental timing cues transmitted from the SCN with the cellular timekeeping system in peripheral organs.

Most timing cues are coordinated and transmitted from the SCN to the periphery. These signals are used by individual cells throughout the body to synchronize their endogenous “molecular clocks”. The molecular clock is formed by a set of transcription factors and transcriptional regulators that generate circadian rhythms with a period of close to 24 h. At the core of the molecular clock are the transcription factors brain and muscle arntl-like1 (BMAL1), a transcriptional activator, that binds with circadian locomotor output cycles kaput (CLOCK) to drive the expression of several genes, including their repressors cryptochrome (CRY1/2) and period (PER1/2/3) which bind together to complete a negative feedback loop. BMAL1, CLOCK, CRY1/2, and PER1/2/3 are considered the core components of the cellular circadian clock loop, and manipulation of the core clock factors can impair cellular circadian rhythms [28,29]. In addition to the core clock loop, there are a myriad of additional factors regulating the molecular clock, including REV-ERBα/β, retinoid-related orphan receptor (RORα/β/γ), D-box binding protein (DBP), cyclin-dependent kinase (CDK1), casein kinase 1/2 (CK1/2), and F-box/LRR-repeat protein 3 (FBXL3), together influencing the core molecular clock to adjust the pacing of cellular timekeeping (see review [30]). To time specific functions within cells and tissues, the molecular clock controls a subset (~15–25%) of cell-specific genes, termed clock-controlled genes [31]. As clock-controlled genes are under the control of the molecular clock, they exhibit a ~24 h expression pattern, which allow the optimization of cellular functions [32,33,34,35]. 

### 1.2. Female Reproductive Success Requires Precise Coordination of Hormone Release Patterns

Female reproductive success requires the precise coordination of hormone release patterns for pregnancy to occur, including ovarian follicular development and maturation, ovulation, implantation, pregnancy maintenance, labor, and birth, as reviewed in [36]. Each of these processes require specific coordination of a combination of hormone signals, which form part of complex feedback loops, including the regulation of the hypothalamic–pituitary–ovarian (HPO) axis. At the apex of the HPO axis are the kisspeptin and gonadotropin-releasing hormone (GnRH) neurons in the hypothalamus. Direct and indirect projections from the SCN are required for timed hormone release and fertility [37,38,39,40,41,42,43]. To regulate pulsatile luteinizing hormone (LH) and follicle stimulating hormone (FSH) release from the pituitary gonadotrophs, appropriate GnRH pulsatile release at the median eminence is necessary. Temporal aspects of GnRH pulse frequency regulate the downstream hormone release, by which increased GnRH pulse frequency favors LH expression [44,45] and decreased GnRH pulse frequency favors FSH expression [46,47,48]. LH and FSH promote gametogenesis and gonadal sex steroid release, allowing gonadal sex-steroid feedback onto the hypothalamus and pituitary [49]. GnRH release patterns are primarily controlled through sexually dimorphic kisspeptin neuron projections onto GnRH cell bodies and terminals. Kisspeptin-expressing neurons located in the anterior ventral periventricular area are involved in the LH surge and account for a larger neuronal population in females than males. The arcuate nucleus kisspeptin neurons are important in mediating the estrogen-negative feedback onto GnRH neurons and are central in generating pulsatile GnRH release [50,51]. The fine-tuning of these hormone release patterns is supported by the molecular clock within reproductive tissues, where cell-endogenous clock function has been shown to be important for ovarian steroidogenesis [52,53], ovulation [32,54], implantation [55,56] as well as pregnancy and parturition [43,57]. As the timing and synchronization of hormone release plays an essential role in fertility, the desynchrony between the SCN and the rest of the HPO axis is a potential contributor to reduced reproductive success found in clinical studies of shiftwork.

In addition to the reproductive hormones mentioned above, one of the best-known circadian hormones, melatonin, frequently referred to as the “hormone of the night”, plays important roles for both circadian timekeeping and female fertility. Melatonin plays numerous roles in the body, where its circadian release is known to translate day-length information to the body [58] and its potent antioxidant effects [59] play important roles in pregnancy [60]. Specifically for this review, the role of melatonin as a sleep promoter [61,62] is of relevance as shift workers tend to experience poor sleep quality, a problem that could be improved through exogenous melatonin consumption [63]. The antioxidant effects of melatonin are important to consider during pregnancy, where nocturnal levels of melatonin increases [60]. During pregnancy, melatonin is thought to play a protective role for the developing fetus by reducing oxidative stress [59]. For more information about the role of melatonin in pregnancy, recent work has thoroughly reviewed this topic [64]. Importantly, melatonin release is regulated by light, as it is released by the pineal gland in darkness. Pineal melatonin release is suppressed by light at night, making it of special interest to consider as a mediator of the adverse effects of nighttime and shiftwork. Although nocturnal melatonin release is systematically repressed by white (as well as green and blue) light, a large variation in sensitivity to the suppressive effect of light on melatonin exists in humans [65], providing one mechanism explaining the great variation in physiological responses to light at night and shiftwork in humans. Of additional note, while an in-depth examination of potential therapeutic targets for shift workers is not a focus of this review, the idea of modulating melatonin release via light spectrum modification as a target therapy for shift workers has shown promise in both human [66,67] and rodent [68] models.

### 1.3. Light and Food Timing Can Both Contribute to Circadian Disruption

To date, most studies examining the impact of shiftwork on human physiology have shown an association with endocrine disorders. Correct function of the endocrine system requires the precise timing of hormone release that, when disrupted, often results in disease. Findings from studies of metabolic function consistently show that shiftwork is associated with increased risks of metabolic disorders [19,20] and reduced cardiovascular health [21,22,23], both of which are linked to endocrine disruption [69].

Similar to the vast array of shiftwork schedules in humans, numerous different models have been utilized in laboratory rodents aimed to understand how different aspects of shiftwork in humans, such as exposure to light shifts and mistimed food, are linked to reproduction and fertility. While it is difficult to isolate such variables in studies examining human shift workers, the use of rodent models enables the disassociation of variables known to alter circadian timekeeping, such as light and food. Food timing is a physiological entraining signal by which the body’s circadian rhythms can be adapted [70,71,72,73,74,75]. Disruption of food timing, a common feature of shift work, can also lead to reduced reproductive success in mice. In a mouse model of disrupted food timing, where food was restricted to the light phase (inactive period of the day), it was found that food availability during the inactive phase, but not during the active phase, caused a decrease in copulatory plugs, uterine implantations, and birth rate [76]. This is just one example of the relationships between food timing and how it can impact reproductive function. Although food timing is an important parameter to consider in shift work, it is outside the scope of this review to discuss the role of food timing on physiological functions. For the interested reader, numerous excellent reviews on this topic have recently been published; see reviews [77,78,79,80].

## 2. Chronic Shiftwork Negatively Impacts Fertility in Women

### 2.1. Human Literature Search Criteria

To perform the human literature review, we used a combined NCBI PubMed and Google Scholar search to locate studies published on human shiftwork and reproduction (Table 1). Searches included the keywords: shiftwork, reproduction, fertility, night work, nightshift, rotating shiftwork, hormones, pregnancy, menstrual cycle and labor. Exclusion criteria included requirements for publication in English and operational definitions of shiftwork and/or nightshift, with a focus on primary research papers over meta-analyses. Additionally, the scope of this review includes reproductive and fertility measures up to birth, but does not focus on the long-term influence of shiftwork on offspring, although it should be noted that there is evidence that shiftwork can exert generational effects [81]. Within this review, shiftwork was defined as exposure to any combination of evening shifts, night shifts, and/or rotating shifts. Most commonly, the shifts were split up in day (8 a.m.–4 p.m.), evening (4 p.m.–12 a.m.), and night (12 a.m.–8 a.m.); however, shiftwork schedules varied considerably, with multiple studies having overlapping schedules, 12 or 16 h shifts, limited nightshifts, different cycles of rotation, etc. Shiftwork can be either fixed, where an individual will always be on a certain shift allowing for a regular sleep/wake schedule, or rotating, where employees are rotated between different shifts and are often unable to develop regular sleep/wake cycles. Additionally, rotating shiftwork can be subdivided into two types, constant scheduling, with defined days on and off (i.e., one month on nightshift, followed by two-month dayshift), or irregular scheduling, with no consistently patterned schedule. 

### 2.2. The Menstrual Cycle

**Figure 2:**
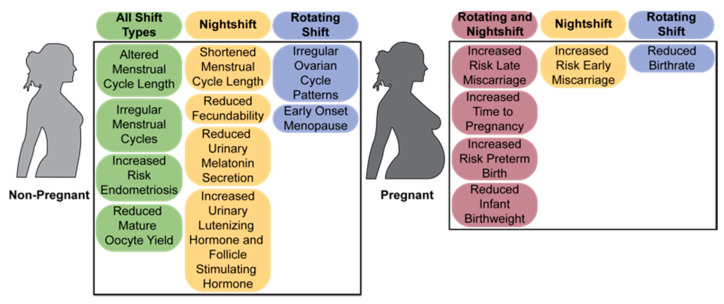
Summary of the impact of shiftwork on reproductive function in women. Effects of shiftwork on female reproduction in non-pregnant (top) and pregnant (bottom) women. Risks associated with different categories of shiftwork are categorized by colors corresponding to the legend at the top, where green indicates all shift types (nightshift, evening shift, and rotating shift), yellow is nightshift, blue is rotating shifts, and purple is both rotating and nightshifts.

The menstrual cycle is particularly sensitive to circadian disruption, as precise hormonal timing and secretion levels are essential for the correct alignment of the positive and negative feedback loops required for normal cycling, as reviewed in [122]. Numerous studies have found that repeated disruption of the circadian timekeeping system in women working nightshift, shiftwork, and rotating shiftwork for extensive time periods experience detrimental effects on the menstrual and ovarian cycles (Figure 2), although not all studies examining the menstrual cycle in shift workers found menstrual disruption [84]. Shiftwork has been associated with altered length and regularity of menstrual [4,5,6,9,82] and ovarian [7] cycles, in addition to the presence of dysmenorrhea (discomfort of the menstrual cycle) [6,83]. In a longitudinal cohort study of 71,077 US nurses, it was found that the probability of irregular menstrual cycles increased in both younger (28–30-year-old) and older (41–45-year-old) women, and the risk of irregular cycles increased for those who maintained rotating schedules for a longer period of time [4], suggesting a possible increase in susceptibility with chronic shiftwork. Though the relationship between duration of shiftwork and irregularity was not explicitly examined, data show that women who have experienced more years of shiftwork have an increased incidence of irregular ovarian patterns [7].

While other studies have not directly drawn lines across chronic versus intermittent shiftwork, menstrual cycle irregularity is consistent across several studies. A study examining working nurses in Taiwan found that workers on the day shift (8 a.m.–4 p.m.) exhibited the highest percentage of regular menstrual cycles, while varying types of shiftwork, including evening shift (4 p.m.–12 a.m.), night shift (12 a.m.–8 a.m.), and rotating shifts (a combination of day, evening, and night shifts), exhibited higher incidence of irregular cycles. This was also supported by studies that found US nurses working shiftwork frequently (53%) reported changes in their menstrual cycle, accompanied by increased dysmenorrhea (18%) [6]. Taiwanese 12 h rotating shift workers exhibited irregular menstrual cycles, although menstrual cycle length and number of menstrual bleeding days did not differ from controls [82], and Chinese nurses [83] and Australian shift workers [85] on multiple types of shiftwork exhibited increased menstrual cycle irregularity. Aside from menstrual cycling irregularity, ovarian cycling (measured by basal body temperature) was also significantly related to shiftwork, in which nurses on rotating shiftwork were more likely to exhibit irregular ovarian cycle patterns and monophasic ovarian cycles, and less likely to exhibit the traditional biphasic temperature pattern associated with ovulation [7]. Repeated light-induced suppression of melatonin combined with light-mediated disruption of circadian rhythms likely contribute to these negative effects on menstrual cycle and follicle maturation. There is evidence that melatonin, LH, and FSH secretion is disrupted in female shift workers [118,119]. In both studies, nightshift workers exhibited reduced urine 6-sulfatoxymelatonin during nightshifts [118,119]. Additionally, in one of the studies, nightshift workers exhibited decreased urine 6-sulfatoxymelatonin during nighttime sleep on off-nights and the decreased 6-sulfatoxymelatonin during nightshift and daytime sleep of night workers was accompanied by increased LH and FSH [118]. This increased LH concentration in night/rotating shifts was also supported in another study [120]. Together, this provides two potential mechanisms by which light at night can impact menstrual cycling and ovarian function. 

In addition to increased risk for abnormal cyclicity, several studies found links between shiftwork and abnormal menstrual cycle length [4,5,6,9]. However, directional impacts (shortening/lengthening) are often reported at similar rates [4,6]. Interestingly, Chung et al., 2005 identified a link between type of shiftwork and its influence on cycle length. Nurses on constant nightshift were more likely to report a shortened cycle length, of which the risk was significantly higher than those on rotating shifts [5], supporting the hypothesis that different types of shiftwork contribute varying risk factors. This hypothesis is also supported by work in a population of Chinese nurses, where rotating shifts were associated with altered cycle length, with no specific directionality, but frequency of nightshifts was associated with menstrual cycle shortening [83]. However, there was an additional study in Italian shift workers that determined a link between shortened estrous cycles and rotating shift work [9]. Further, two studies on Taiwanese nurses working in the ward, emergency room, and intensive care unit found that these work environments were more likely to cause irregular cycles compared to nurses in the outpatient department and operating room [5,7]. The wards, emergency room, and intensive care unit consisted of rotating shifts while outpatient department nurses had day shifts; the operating room shifts were not specified [5]. Additionally, the amount of rotating shiftwork varied as some studies had continuous rotations [5,7], while others had as little as three nights of rotations per month [4]. These differences in work schedules could be the cause of discrepancies in results.

Shiftwork is known to disrupt sleep by causing an increased number of awakenings and decrease the overall sleep length [6]. Decreases in sleep can lead to sleep deprivation among shift workers, which alters hormone levels. LH, estriol, and thyroid-stimulating hormone have been shown to increase from partial sleep deprivation, with thyroid-stimulating hormone increasing significantly and remaining at high levels following sleep deprivation [117]. Further, light at night changes cortisol levels, indicating light can modulate the stress axis [123]. Indeed, some shiftwork studies have noted a correlation between increased stress and menstrual abnormalities, such as delayed menstruation [8]. Many shift workers report an increase in perceived stress relating to working conditions and responsibilities [7]. One study provided a questionnaire that collected information on perceived work stress of female nurses, finding that self-reported work stress was most commonly reported in nurses working in wards and intensive care units, compared to other hospital sectors [5]. While this study found no correlation with stress and changes in the menstrual cycle, another study found that nurses in high stress units had an increased risk of long menstrual cycles [9]. This association of increased work stress and changes in menstrual cycle was supported by two other studies that found a high prevalence of dysmenorrhea accompanying the menstrual cycle [124] and increased likelihood of menstrual dysfunction [125] for female workers who experienced elevated work stress. This suggests a likely interaction between stress and shiftwork, which should be further examined in future studies. Given the wide consensus of shiftwork increasing the risk of irregular menstrual cycling, it is clear that working shift schedules presents a high risk of disruption to the female reproductive cycle, which could present further complications for fertility. 

### 2.3. Fertility and Miscarriage

In addition to increased risk for disrupted menstrual cycling, shiftwork has been linked to a reduced childbearing rate [10], reduced fecundability (the probability of conceiving in a given month of a pregnancy attempt) [88], and increased time to pregnancy [87,90] (Figure 2). Specifically, rotating shiftwork was determined to decrease childbearing rates with rotating shift workers experiencing a significant reduction in childbearing rate compared to women working a consistent daytime schedule [10]. Rotating shiftwork [87] as well as evening and nightshifts [90] were associated with requiring a longer time to become pregnant compared to daytime workers and African American nightshift workers exhibited reduced fecundability by 20% compared to those who had never worked a nightshift [88]. To note, others did not find a correlation between any type of shiftwork on duration of pregnancy attempt, which was used as an indirect measure of fecundability [89]. In addition to decreased childbearing rates and fecundability, young shift workers are more likely to required fertility treatment to conceive [85]. The influence of shiftwork extends past requiring fertility treatments [85] and has been documented to influence the success of fertility treatments [86]. In a population of women attending an academic fertility treatment center, reduced mature oocyte yields have been documented in women working evening/night/rotating shifts compared to day shift workers [86]. One possible explanation for the decreased childbearing rate for rotating shift workers is that it could be linked with disruptions found in the menstrual cycles of shift workers [4,5,6] via hormone disruption [126]. This is supported by the inverse relationship between nights worked and melatonin levels, where night work reduced melatonin levels [89,90,102] and increasing the number of rotating night shifts decreased melatonin levels [115,116]. Further, rotating shiftwork is associated with increased estradiol [115,116,121], whereas no impact or an increase on progesterone levels has been observed in these same populations [115,116]. As correct hormonal changes are required for the menstrual and ovarian cycle, such increases in estradiol, and potentially in progesterone, might impact target tissues function and can cause either a desensitization of target receptors (reducing the sensitivity of target tissue to the hormones), or a promotion of positive feedback loops, increasing target tissue responses to the hormones. Either way, the increased levels of sex steroids would be expected to deregulate hormonal feedback mechanisms regulating the menstrual cycle [127].

Although aberrant menstrual and ovulatory cycles are expected to impact fertility and reduce childbirth rates, there are several other factors that could account for or contribute to fertility deficits in shift workers, including an increased risk of spontaneous abortion, or miscarriage (Figure 2) [11,12,13,94]. Early miscarriage, between weeks 4 and 13 of pregnancy, was assessed in a study that compared US flight attendants, evaluated by flight schedules compared to home time zone, and teachers, who had normal working hours [11]. This study found no increase in the likelihood of miscarriage for flight attendants compared to teachers. However, within the flight attendant group, working during the normal sleeping period (10 p.m.–8 a.m.) increased the risk of miscarriage [11], suggesting that sleep and/or circadian disruption during the first trimester of pregnancy could play an important role in pregnancy maintenance. It is important to note that early pregnancy loss (<12 weeks) often remains undetected and therefore is difficult to evaluate accurately. Even with the difficulties in assessing early pregnancy loss, there are still links that suggest shiftwork during early pregnancy is a risk factor for miscarriage. According to a study assessing the relationship between spontaneous abortion (miscarriage), and work schedules of US nurses, women working nightshifts had a 60% increased risk of first trimester miscarriage compared to women working days only, but there were no increased risks for rotating shift schedules [12]. In the same study, late (12–20 weeks) spontaneous abortions were more prevalent in women who rotated day and evening shifts than daytime shifts [12]. These increases in risk of pregnancy loss were supported by additional studies examining combined evening, night, and rotating shiftwork which found an increased risk for miscarriage over fixed daytime schedule women [92] as well as an increased risk for early [91,93] and late [95] miscarriage with nightshift workers. Interestingly, the risk of pregnancy failure appears to increase with chronic shiftwork, as a Danish nationwide cohort study evaluated the influence of nighttime work the week prior to miscarriage and found increased risk of miscarriage after week 8 of pregnancy if workers were exposed to two or more night shifts the previous week. However, there was a cumulative effect of nightshifts on pregnancy, where the risk for miscarriage increased in a dose-dependent manner following multiple weeks of nightshifts [13].

Potential hormonal mechanisms, which could be deregulated in shift workers, are the levels of progesterone, melatonin, or uterine sensitivity to hormones. Progesterone, an ovarian produced hormone (Figure 1), is required for implantation through its action on the endometrium in the uterus [128]. Non-pregnant women working rotating shifts had no [115] or a modest increase [116] in progesterone. Such an increase would be expected to favor implantation. However, more studies are needed to assess how progesterone levels change in pregnancy during shiftwork before we can determine whether shiftwork associated changes in progesterone might cause early pregnancy loss. Melatonin may also play a role given its importance during pregnancy [64] in conjunction with evidence from shift workers that indicate nightshifts reduce pregnancy nighttime urine excretion of 6-sulfatoxymelatonin [119]. If shiftwork-induced changes in hormone release are not causing early pregnancy failure, light-induced changes in circadian rhythms at the level of the uterus might. Recent work in humans and human cell models have associated the molecular clock gene BMAL1 in decidualization, a process involving a rapid morphological and functional differentiation of the endometrium allowing implantation [129]. Future studies further exploring light-induced disruption of uterine function and clock gene expression will be essential to understand the role of BMAL1 in implantation and determine whether the increased risk of miscarriage during shiftwork might be associated with abnormal molecular clock function in the uterus.

### 2.4. Labor and Birth

Although shift workers are at higher risk for miscarriage, most shift workers give birth successfully. That said, several studies indicate shift working mothers are at significant increased risk for both early (before 37 weeks of pregnancy) [16,18,97,102,105,107] and late birth (after 42 weeks of pregnancy) [17], although several studies found no significant relationships between shiftwork and preterm birth [96,103,104,106]. Gestation length plays an important role in fetal development and maturation. In humans, a baby is considered at term when delivered between 38 and 42 weeks of pregnancy. Preterm birth (before 37 weeks) is associated with an increased risk in infant mortality [130] and is often associated with sever developmental delays, including smaller size and immature or improper development of the respiratory, cardiovascular, and central nervous system [131]. A negative impact on fetal wellbeing is further supported by a small series of case studies showing reduced Apgar scores of babies from nightshift working mothers, which was associated with reduced maternal melatonin in the third trimester of pregnancy [132]. Further, one study showed that rotating shift workers in Chinese textile mills were more likely to experience preterm birth (20%) compared to regular scheduled workers (15.1%), and the incidence of preterm birth increased slightly when production workers only were analyzed [16]. Another study of Swedish midwives found that the rate of preterm birth increased for nightshifts (9.1%) and rotating shiftwork (three-shifts, 4.6%) compared to that of day shifts (3.5%) [18]. An additional study of Canadian women found increased risk of preterm birth in rotating shift workers [97]. In contrast, a Danish study found that rotating shiftwork had no association with preterm birth and that fixed night workers had a high risk of post-term birth [17]. Post-term birth (after 42 weeks) has been associated with an increased infant mortality [133]; however, less is known about the long-term effects on development. Recent studies have shown an association between post-term delivery and emotional and behavioral problems in early infancy [134]. It was noted in the shiftwork study that the result of no association with preterm birth varied from most studies and was attributed to the fact that in Denmark shift workers had shorter working hours and more free time [17]. 

Labor onset is regulated by a myriad of factors, including circadian timekeeping and light. Thus, the disruption of the circadian system caused by extensive light at night in shift workers might be a contributing factor to the increased risk of mistimed birth in this population. Disrupted sleep is a common problem in nurses working shiftwork, which frequently report having a significant number of awakenings from sleep (*p* < 0.001), as well as a decrease in sleep length (*p* < 0.001) after working night shift rather than day or evening shift [6]. Interestingly, the timing of birth in a large number of species, including humans and rodents, coincides with the wake-sleep cycle, where birth rates increase significantly during the rest and sleep phase [135,136,137]. The role of light and circadian rhythms in labor is further supported by an observational study of women in their ninth month of pregnancy, where sleeping less than 6 h per night was associated with significantly longer labors, as compared to women who slept more than 6 h per night. In addition, the “short sleep” group was 4.5 times more likely to have a C-section compared to women who averaged more than 6 h of sleep [14]. A role of circadian rhythms/sleep in labor progression is further supported by a study of women with severe sleep disturbance, who presented with significantly longer labors and were 5.2 times more likely to have a C-section [14]. Thus, sleep disruption in shift workers could be an important variable to study in pregnant shift workers. 

In addition to disrupted sleep, nocturnal light-induced changes in hormone release might be an additional contributing factor in regulating labor. One hormone thought to be involved in promoting nocturnal labor and birth is the nighttime release of melatonin [137,138,139,140,141], a hormone which becomes increasingly released during pregnancy [142] and potentiates oxytocin-induced uterine contractions [143,144]. Light at night suppresses melatonin, which is associated with reduced uterine contractions in term-pregnant women [144,145,146]. Interestingly, in vitro studies show that in the human myometrium melatonin receptor is a clock-controlled gene, and its expression is directly regulated by BMAL1 [147]. Of note, a very small cohort study found a premature increase in the expression of the melatonin receptor in the uterus of women having preterm birth [137]. The modulatory role of melatonin on uterine contractions is further supported by the finding that labor duration is significantly longer when women labor during the day (no pineal melatonin), versus the night (release of pineal melatonin) [148]. The influence of hormones and disrupted sleep are not mutually exclusive, and it is likely that the contributions of sleep and related hormone disruption are interlinked. 

A growing body of literature suggests that hormone imbalance during pregnancy likely results in negative fetal outcomes, including various negative health outcomes later in life (Table 1). Multiple studies determined an increased risk of low birthweight in shift working mothers [10,16,96,97,98,99,100,101]. At a Taiwanese semiconductor factory, it was found that newborns of women working persistent shift work schedules had a reduced mean weight of 2998.5 g compared to that of babies born to intermittent shift workers, which weighed an average of 3251.3 g, and daytime workers, with an average wight of 3271.7 g [10]. Additionally, women exposed to persistent shiftwork schedules birthed newborns an average of 250 g lighter than women working other types of shift schedules, and 35% of the babies born by persistent shift workers fell within the lightest quintile birth weight (1950–2834 g) [10]. It is also important to note that the exposure to chemicals within the semiconductor factory and its effect on birthweight was not examined further within this study. Despite this limit, the negative impact on birthweight identified in the previous study is supported by several studies examining rotating and nighttime shiftwork that found increased risk of low infant birthweight [16,96,97,98,99,100,101]. Interestingly, a Danish study found that fixed night shifts slightly decreased birthweight by a mean of 30 g, while rotating shiftwork did not significantly impact weight [17]. These differences might be due to differences in work schedules as rotating shiftwork in Denmark limits night shifts for workers (average 4 nights per month), compared to the Taiwan semiconductor factory, where women worked a persistent rotating shiftwork schedule (6 days on, 3 off, 6 nights on, 3 off) or the textile workers, who had a continuous eight-day cycle of shifts [10,15,16,17]. During fetal development, changes in maternal hormone release patterns should also be considered as potentially impacting fetal growth and maturation [149]. The ovarian steroids, estradiol, and progesterone have been found to be positively associated with birth weight in the later weeks of pregnancy [150]. As previously mentioned, non-pregnant shift workers, have been shown to have normal [115], or increased progesterone levels [116], and increased estradiol levels [115,116]. These increased levels of progesterone and estradiol would be expected to promote fetal growth, and thus could play a protective role counteracting the negative impact on fetal growth in women working shiftwork. 

### 2.5. Menopause and Reproductive Disorders

Various other health and reproductive disorders are found to have an association with shiftwork, though extensive examination of these topics lie outside the scope of this review. This section aims to provide a brief overview of the influence of shiftwork on the ageing reproductive system and risk of reproductive disease pathology. 

Menopause is the biological process that marks the end of a woman’s menstrual cycles and is a period associated with great hormonal fluctuations. Although women can go through menopause as early as their 30s and 40s, the median age of menopause in the US is 51.3 years of age [151]. Menopause is associated with a wide variety of physical symptoms, such as hot flashes, disrupted sleep, lower energy, and reduced emotional health [151]. It is well established that the onset of menopause later in life is linked with multiple positive health outcomes including a decreased risk of developing cardiovascular disease and an increase in life expectancy [152]. A 22 year follow-up cohort study on US nurses determined that rotating shift workers presented an increased risk of early menopause, with women under the age of 45 having an increased likelihood for early onset [108]. Furthermore, women under the age of 45 who worked rotating shifts for 11–20+ years presented a 22% increase in risk for early menopause [108]. Thus, shiftwork can be considered an important risk factor advancing the age of menopause in women.

Shiftwork has now repeatedly been associated with increased cancer risk [153], including an increased risk of developing breast and ovarian cancers. A study of US nurses found that premenopausal women who worked rotating shifts for 1–14 years saw a 23% increased risk of breast cancer [109]. The same study found that postmenopausal women who worked rotating shifts for 30+ years had a significantly increased risk for breast cancer [109], suggesting a potential interaction with advanced reproductive age and breast cancer risk. Additional studies determined an increased risk between shiftwork and ovarian cancer [111,112], while others show no association [113], creating a need for additional work to investigate mechanisms behind this discrepancy. Furthermore, the risk of developing endometrial cancer, cancer within the uterine tissue, is significantly increased in women who have worked shiftwork for 20+ years, a risk that more than doubles for women working shiftwork with a body mass index of 30 or greater [110]. In addition to cancer, endometriosis, a reproductive disorder characterized by growth of uterine tissue outside of the uterus, is commonly accompanied by excruciating and debilitating episodes of pain. One study found that there was a 50% increase in the possibility of developing endometriosis with any type of night shift work and this risk doubled when women worked more than half of the shifts at night [114]. This increased risk for endometriosis has also been documented in a population of Southern Australian female shift workers [85]. It is possible that women in shiftwork are even more susceptible to cancer, as endometriosis has been shown to increase the risk of developing various cancers including ovarian cancer, breast cancer, and hematopoietic cancers [154]. Major health risks regarding reproductive disorders and the health of reproductive tissues have been seen to be greatly impacted by shiftwork. Other health risks, such as ovarian cancer, have only begun to be examined in recent studies. 

## 3. Rodent Models of Shiftwork

### 3.1. Methods of Evaluating Rodent Light-Shift Studies

**Figure 3:**
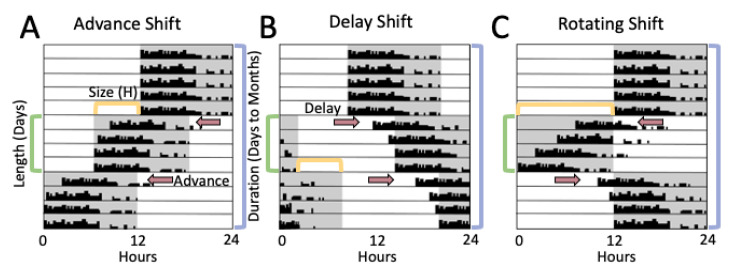
Commonly used light shift paradigms in rodent study models. Single plotted actogram examples of rodent locomotor activity to describe methods commonly utilized to evaluate the influence of light shifts. Shaded areas indicate time of lights OFF, and white areas indicate time of lights ON over a 24 h day. Three main types of light-shift paradigms separated by shift direction, with advancing shifts (**A**), delaying shifts (**B**), and (**C**) rotating combination of advancing and delaying shifts. Other variables important to light-shift paradigms include shift size (time in hours (H) of the shift, typically ranging between 3 and 12 h), shift length (number of days between shifts, indicated by the green brackets), and shift duration (number of days exposed to the shift paradigm, indicated by blue brackets). Arrows indicate day and direction of shift.

To perform the rodent literature review, we used a combined NCBI PubMed and Google Scholar search to locate studies published on rodent models of light shifts and reproduction (Table 2). Searches included the keywords: shiftwork, reproduction, fertility, laboratory rodent, mouse, rat, hamster, hormones, light shift, pregnancy, estrous cycle, and labor. Studies in this review were focused to rodent paradigms that evaluate reproductive function and rely on the influence of mistimed light, although there are several other methodological paradigms that mimic other aspects of shiftwork, such as food timing and disrupted sleep [78]. Following determination of the studies for inclusion, there were several different rodent models of light shifting, as there does not exist a standard paradigm in the field, and rarely are the same models utilized across laboratory groups. To compare across different light shifting schemes, we focused on four major aspects by which lighting schemes can be organized. The four major aspects (Figure 3) include:

1. Direction. Three types of light schedules are typically studied: light advance (lights are turned ON earlier than previous cycle), light delay (lights are turned OFF later than previous cycle), or rotating, in which both advances and delays are incorporated into the model.

2. Size. The shift size is the number of hours the new lighting schedule is different from the original light schedule. This includes a variety of shift sizes, commonly including 6, 10, and 12 h shifts, in which 12 h shifts are often referred to as reversals.

3. Length. The shift length is the number of days between shifts. 

4. Duration. The duration of the shift is the time course of the shift paradigm, or the number of days to months that the schedule is utilized.

### 3.2. Light Shifts Rodent Models and Estrous Cycling

Laboratory rodents do not experience menstruation, but progress through cyclical patterns of reproductive physiological changes called estrous cycles. A number of studies have evaluated the influence of shifting light on rodent estrous cycling, with results dependent on both shift size and shift duration. One study directly evaluated the influence of several different shift reversal patterns on estrous cycling in mice, specifically focusing on shift length [157]. This work focused on three different experimental schedules, all of which incorporated full 12 h light reversals, but varied by the number of days (3 day, 6 day, or 12 day) between light reversals. Estrous smear data indicated that 3 day 12 h light:12 h dark (LD) rotations caused more severe disruption to cyclicity than the 6 day rotations, whereas the 12 day group experienced the least number of mice with irregular estrous cycles. Interestingly, estrous cycle length was increased in all shift groups compared to stable light controls [157]. These findings are unsurprising, as decreased light shift lengths (few days between shifts) is thought to be more disruptive to circadian timekeeping in the SCN which quickly adapts to the photic cues, while cellular and hormone release patterns require more time to adjust to a novel lighting schedule. More work focused on tissue timekeeping and hormone release following different shift lengths will be necessary to fully understand this relationship.

While the shift length appears to play an important role in the degree of estrous cycle disruption, additional work suggests that shift direction also determines the influence of lighting changes on rodent models. One mouse study determined that chronic rotating shifts, with 10 h rotating shifts every 3–4 days for up to 9 months, increased the likelihood of developing irregular estrous cycles, with no changes in estrous cycle length and increased incidences of acyclicity over time [155]. Importantly, this study examined the influence of a single phase delay or advance of light on estrous cyclicity in addition to chronic shifting and determined that a single shift was not enough to induct acyclicity. However, both the singular advancing and delaying light shifts increased the length of the second estrous cycle and increased the intraindividual variability of the second and third estrous cycle following the shift [155]. In addition to estrous cyclicity, the influence of phase shifts on the timing of the LH surge was assessed in both singular and chronic light shifts. For singular light shifts, both advancing and delaying shifts reduced the number of mice exhibiting an LH surge and altered the timing of the LH surge, in which the surge for both the advance and delay occurred ~3 h earlier than the control group [155]. This change of the LH surge timing following a photic phase shift is consistent with effects on the LH surge in hamsters, where single 3 h phase advances and delays result in altered timing, shape, and magnitude of the LH surge, although the LH surge was not abolished and there was a distinct direction dependence in hamsters that was not present in the mouse model [163]. These differences may be due to shift size, where the mice received 10 h shifts, compared to the less disruptive 3 h shifts in hamsters. Another possibility is a potential modulatory role of melatonin in LH surge adaptation to light shifting, where the mouse model used in the study [155], is known to be melatonin deficient [164], whereas hamsters produce pineal melatonin.

In addition to shift size, the duration of shifts seems to play an important role on the impact of lighting shifts on the estrous cycle, by which chronic shifting increases the disruptive effect. In the mouse model with chronic 10 h rotating shifts every 3–4 days, estrous was evaluated for up to 9 months, with increased incidences of acyclicity over time [155]. Further, these rotating shifts alter the pattern and amplitude of the LH surge where the mice displayed a reduced preovulatory LH surge at several time points throughout the duration of light shifts, including at the beginning of the shift paradigm, and after 6 months, and 9 months of light shifts, indicating underlying hormone disruption likely contributes to reproductive cycle dysfunction. 

Ageing may play a role in the susceptibility to reproductive cycle deficits following light shifts. Ageing weakens the circadian timekeeping system [165,166,167], and rodents with circadian clock gene mutations have been linked to early reproductive senescence [43,168]. Indeed there is evidence that light shifts are more disruptive with age, as weekly intermittent shifts have been shown to increase the irregularity of estrous cycling in middle-aged (8–12 month) female mice, but not young females (2–6 month) [156]. This age-related increase in estrous cycle disruption following light shifting is also supported by work in knock-out (KO) mouse models, specifically deficient of the clock genes Cry1 and Cry2 (*Cry1/2* KO). Both *Cry1* KO and *Cry2* KO mice exhibited irregular estrous cycle patterns, which were characterized by vaginal cytology presenting with more days with increased cornified cells (3 days) typically seen during the transition to reproductive senescence [156]. This work may indicate an interplay between light shifts, age, and cellular clock function, although more research is needed to understand how these variables may alter reproductive health. 

### 3.3. Light-Shift Rodent Models, Pregnancy, and Labor 

In rodents, like humans, implantation and pregnancy maintenance are regulated by the circadian timekeeping system (Table 2). Several studies have found evidence that light shifts can impact pregnancy rates [155,162], litter size [155], and successful birth rates [155,162], in addition to hormone and clock gene expression [161], although many of these results differ by model. Similar to studies examining estrous cycling, light shift paradigms focused on pregnancy appear to be dependent on the direction of light shifts. Work by Summa et al. evaluated the influence of the direction of light shifting utilizing 6 h phase shifts (either delaying or advancing) every 5–6 days during pregnancy. Pregnancy success was decreased greatly in both shift conditions, in which the phase advance (22% success) and delay (50% success) both had decreased pregnancy success compared to controls [162]. Notably, phase advances caused a greater disruption on pregnancy success than delays, which is consistent with several studies indicating that mice exhibit greater adaptive entrainment responses to delaying light pulses over advancing pulses [169,170,171]. Disruption to pregnancy success was also found in mice experiencing chronic rotating shifts (10 h advance followed by 10 h delay every 3–4 days) for 4 weeks prior to mating. These chronic rotating shifts reduced the number of mice that carried pregnancy to term, number of pups per litter and overall number of pups, and the rotating shift group was less likely than controls to give birth between 20 and 24 days after mating [155]. Interestingly, such disruptions to pregnancy rates were not present in rats exposed to full light reversal (rotating) phase shifts every 3–4 days during pregnancy, where no alterations to gestation length, litter size, survival to weaning, or birth rate were noted [160]. It is possible that these differences could be due to rodent model (rat versus mouse); however, it should be noted that both mouse studies utilized the same strain, C57/Bl6J, which do no not secrete melatonin [164], a hormone that has been suggested to be involved in litter size, fetal growth and is able to counteract light-induced reductions in implantation [172,173]. Interestingly, dam melatonin secretion was not altered following light shifts during rat pregnancy [161], as hormone levels and circadian rhythmicity remained similar to controls. Given melatonin’s important role in pregnancy (see reviews [58,64,137]), it is possible that the absence of this hormone may weaken the circadian system, leading to an increased risk of circadian disruption following light shifts, although much more work is needed to evaluate this hypothesis. 

In addition to pregnancy success and birth outcomes, there is evidence that light shifts can alter hormone and metabolite concentrations in pregnant dams. Specifically, in the rat model, rotating 12 h phase shifts every 3–4 days during pregnancy disrupted rhythms of corticosterone, which was not rhythmic in shifted mice, in addition to altered rhythms of several metabolites, including glucose, insulin, plasma leptin, and free fatty acid concentrations [161]. In addition to the 24 h analyses of hormones and metabolites, this model also evaluated maternal liver clock gene expression, finding that rotating shifts disrupted the rhythmicity of Bmal1, Per1, Per2, and Rev-erba mRNA [161]. The disrupted liver tissue rhythmicity likely indicates further disruption of circadian timekeeping in peripheral tissues. While more investigation is needed to determine whether reproductive tissue timekeeping is disrupted following light shifts, it is becoming clear that the molecular clock plays a role in the uterus for both implantation and labor onset. Indeed, studies in rats and mice have shown that the non-pregnant uterus possesses a functional molecular clock [174,175,176], which becomes upregulated during pregnancy, and the circadian reporter mouse *Per2*::luciferase, which reflects on the molecular clock function within cells, adapts a longer circadian period in the uterus towards late pregnancy [176], although the functional consequence of this slower running circadian clock remains unknown. The involvement of the molecular clock in both pregnancy maintenance, labor and birth is further supported by studies in transgenic mice where *Bmal1*, *ClockΔ19*, *Per1*, and *Per2* mutant females have delayed and reduced embryo implantation, increased embryo resorption, and when they do become pregnant, often produce smaller litters [43,56,168,177,178,179]. In the case of the *Bmal1* KO females, the lack of implantation is not only caused by malfunction of the uterus, but can be partially contributed to the loss of the ovarian production of progesterone. Indeed, 38% of *Bmal1* KO females develop implantation sites when receiving a daily progesterone injection from post-coital day 3.5 till 10.5 [35]. This leaves ~60% of *Bmal1* KO females supplemented with progesterone without implantation sites, supporting recent work involving *Bmal1* in the endometrium for embryo implantation in rodents and decidualization in humans [180,181]. Taken together, both rodent models of genetic weakening of circadian rhythms, as well as models of light-induced circadian disruption point to an important role of circadian timekeeping in reproductive function and show that normal pregnancy requires an intact molecular clock, and proper physiological entrainment to the day-night cycle. 

## 4. Discussion

### 4.1. Connections and Limitations between Human Studies and Rodent Light-Shift Models

From the combination of work above, there are several connections and distinct differences between data generated from human shift workers and rodent light-shift models aimed at understanding how aspects of shiftwork function to produce reproductive deficits. It is important to note that both human and rodent studies contain limitations, which alone make drawing conclusions about the nature of shiftwork difficult. 

While assessing the influence of shiftwork on the female reproductive system is the most direct way to understand health risks of shift workers, there are several difficulties with such work. Although work to date has provided an excellent foundation in this topic, perhaps the biggest drawbacks in the current body of human shiftwork literature are the correlational nature and reliance on subject self-reporting utilized in much of the work. Indeed, many studies, including longitudinal studies of shift workers, suffer from difficulties with subject retention and control over experimental variables. While human experimental setups in which several physiological variables, such as ovulation, hormone release, and tissue samples, could be collected and shift schedules could be experimentally controlled, such studies are subject to higher ethical scrutiny and, with increases in participant burdens, often come decreases in subject recruitment and retention. However, even with such difficulties, a number of human studies have been successfully completed and contributed greatly to our understanding of reproductive health risks of shift work. 

While the building of statistical models in clinical studies can aid to piece apart the contributions of different health risks, studies in laboratory rodents are able to experimentally isolate the contributions of variables both in aspects of shiftwork and their reproductive outcomes, often resulting in more specific and mechanistic insights. Even with the ability to generate highly specific mechanistic answers to hypotheses, laboratory rodent studies are not without their own limits. Importantly, while laboratory rodent models are often grouped, there are distinct differences between species (even the nocturnal rodent species most commonly used in circadian research, including the hamster, rat, and mouse), in addition to strain differences within a species. While the circadian system is highly conserved between species, there are several differences between species [182], as well as documented strain differences [183,184,185], which must be taken into account when comparing studies. One such difference is in melatonin synthesis, which plays important roles in both circadian timekeeping and reproduction. The most commonly used mouse strain, Bl/6J, do not secrete melatonin [164]. However, other mouse strains, rats, and hamsters have endogenous melatonin release. Even with these limitations, the combination of both types of studies, human and rodent models, in addition to the ever-growing body of work examining the influence of shiftwork on female reproduction are leading to significant leaps toward understanding and preventing such effects. 

### 4.2. Menstrual Cycle/Estrous Cycling

Mammals, including humans and laboratory rodents share many similarities within the reproductive system, including the HPO axis and its reliance on the correct timing and level of reproductive hormones (Figure 1). Collectively, human studies have found differing results on the menstrual cycle, dependent on the type and length of shiftwork. Irregularity of the menstrual cycle was evident in all types of shiftwork (Table 1), but the impact of shiftwork on cycle length was less clear (Figure 2). All types of shiftwork altered cycle length, but there are no clear indications of what may influence the direction (shorter versus longer) of such changes in cycle length. Evidence suggests that nightshifts may be more likely to shorten menstrual cycle length, but this was not consistent across studies (Table 1). In rodents, alterations to the estrous cycle were found following shift paradigms, although this was also not consistent across models (Table 2). A significant step towards improving the mechanistic understanding of how light shifts may disrupt estrous cycling was evaluated in a light-shift mouse model, through the evaluation of both estrous cycling and LH release patterns [155]. It was determined that both advancing and delaying light shifts alter the timing of the LH surge in a direction-specific manner [155], providing a potential mechanism by which adaptations to light shift can alter hormone release and disrupt cycling and ovulation. It will be important to determine whether this effect is persistent in human populations and if such changes to the LH surge are also prevalent across different types of shiftwork. Additionally, both human and rodent data suggest that lengthened exposure to shiftwork (or light shifts) leads the increased vulnerability to disrupted cycling. Important next steps to understanding the influence of chronic shiftwork on the female reproductive cycle include identifying how hormone changes and tissue timekeeping adapt or react to differing shift durations, ideally in both humans and rodents. 

### 4.3. Pregnancy and Labor

In addition to the ability of rodent light-shift studies to recapitulate clinical data focused on reproductive cycling, rodent models of pregnancy also find several similarities to health risks in human shift workers. Human studies of shift workers identify health risks of several aspects of pregnancy through labor, from early miscarriage to abnormal gestation lengths and reduced birthweights (Table 1). A variety of rodent light-shift models identified several similar findings to human shift workers, including reduced pregnancy rates and decreased live births, although these findings are inconsistent across models (Table 2). While decreased pregnancy success and abnormal gestation lengths were commonly found in human shift workers [10,16,17,18] and mouse light shift paradigms [155,162], this was not the case in a rat light-shift model [161]. While differences in model design, including differences between the four parameters of light shift that were utilized (Figure 3) or species may underlie these discrepancies, one potential explanation that merits further review is hormonal changes in these models. There are strong indications that hormone disruption, specifically melatonin, may play an important modulatory role in the degree to which shift work and light shifts disrupt pregnancy. Both mouse studies evaluating light shifts and pregnancy utilized the same strain, C57Bl/6J, which lacks endogenous melatonin [164], and notably, in the rat model, rotating light shifts during pregnancy did not disrupt dam melatonin rhythms [161]. Taken together, these results suggest a potential modulatory role for melatonin on pregnancy success and gestation length. 

Another discrepancy between human shift workers and rodent models of light shift is birthweight. While low birthweight emerges consistently as a risk for offspring of female shift workers (Table 1), this was not evident in rodent light-shift models (Table 2). Given the absence of birthweight alterations in rodent models of light shift, it is possible that light may not be the variable driving low birthweights in the clinical shift worker population. Another possibility is that human shift workers have a more pronounced mistiming of food intake than rodent models, which might be a contributing risk factor impacting fetal development. Thus, to understand what drives the lower birthweight in shift workers, additional studies focusing on variations within light-shift models and the assessment of other aspects of circadian disruption, such as disrupted sleep and food timing, are needed. More investigation into this topic is necessary and could be important for identifying specific mechanisms by which different aspects of shiftwork may disrupt fetal development and pregnancy.

## 5. Conclusions

A growing body of literature clearly show a negative association between shiftwork and disruption of reproductive function in humans (Figure 2). Thanks to the numerous animal models that have been developed to address different aspects of circadian disruption, either through light-based shift paradigms or genetic weakening of the circadian timekeeping system, a better understanding of the hormonal and functional changes in the body associated with disrupted circadian rhythms is emerging. As current work aims at identifying the functional adaptations in the reproductive axis in response to shiftwork or light shifts, the next steps and challenges will include developing biomarkers to identify individual drivers and underlying causes of shiftwork-induced reproductive impairment, as well as develop efficient treatment strategies to help overcome the negative impact of shiftwork on the increasing population of women working nights and rotating shifts.

## Figures and Tables

**Table 1:** Summary of outcomes in human studies focused on female shift workers and reproduction included in this review.

Publication	Demo-Graphic Location	Mean Age (years)	Study Size (Number of)			Variables	Results
			Rotating Shifts	Night Work	Control		
[4]	United States	28–45	13,349		57,728	Menstrual Cycle Length	Shiftwork associated with cycles <21 d or >40 d
						Menstrual Cycle Regularity	Increased risk of irregular menstrual cycles for women of ages 28–30 and 41–45 and women with continuous rotating schedule
[6]	Midwest United States	29.97 ± 0.6	68	N/A	N/A	Menstrual Cycle Length	22% of shiftwork nurses had a change in menstrual cycle length, including shortened, lengthened, and varied
						Dysmenorrhea	18% of shiftwork nurses had an increase in pain
						Changes in Menstrual Cycle	53% of shiftwork nurses reported some type of change to their menstrual cycle
						Sleep	Decrease in sleep, increase in number of awakenings from sleep, 31% of reported menstrual changes also had increase in sleep disturbances
[5]	Taiwan	27.7 ± 5.3	50	12	72	Menstrual Cycle Regularity	35% of shiftwork nurses had irregular menstrual cycle
						Menstrual Cycle Length	60% of fixed nights shifts and 21.7% of rotating shifts had cycles <25 d, no long cycles
						Dysmenorrhea	30% regularly experienced pain with menstrual cycle
[82]	Taiwan	>17	280		49	Menstrual Cycle Length	Increased risk of short (9.7% vs. 6.0%; *p* = 0.004) and prolonged cycles (26.0% vs. 17.9%; *p* = 0.04) for shiftwork
						Menstrual Cycle Regularity	Increased risk of irregularity (MCL <25 days or >35 days) for shiftwork
[83]	China	21–46	334		139	Menstrual Cycle Length	Significantly higher proportion of nurses with irregularity for shift workers
						Menstrual Cycle Regularity	Having a cycle of 25–31 days decreased from 81.7% to 67.8% after changing to shiftwork
[84]	Spain	<40	113	N/A	75	Menstrual Disorders	No increased risk of having menstrual disorders (duration, dysmenorrhea, excessive bleeding)
[7]	Taiwan	27.7	50	29	72	Ovarian Cycle Regularity	52.6% of rotating shiftwork nurses had irregular ovarian cycle patterns
						Dysmenorrhea	30% had regular dysmenorrhea
[10]	Taiwan	Daytime Work: 26.7 ± 6.3, Intermittent Rotating Work: 30.2 ± 8.7, Persistent Rotating Work: 27.5 ± 8.5	303	N/A	137	Childbearing Rate	Decrease in childbearing rates for rotating shift workers
						Birthweight	Persistent rotating work decreased birth weight by 250 g. Thirty five percent of babies born were in the lightest quintile birth weight (1950–2834 g)
[85]	Australia	N/A	N/A	11,000	84,991	Fertility	Night shift workers ≤35 years more likely to require fertility treatment, no associations among women >35 years; among women who did fertility treatment, night shift workers were more likely than day workers to have menstrual irregularity or endometriosis
[86]	United States	35	36		426	Fertility	Evening/night/rotating shifts had close to 25% less mature oocytes
[87]	Sweden	<29–35+	457	100	189	Fertility	Night shift, two-shift, or three-shift rotation workers had decreased fertility
[88]	United States	34.9 ± 2.7	N/A	196	364	Fecundity	Women who reported ever working night shifts had 20% lower fecundability compared with those who never reported night shift work
[89]	United States and Canada	33	344	298	1006	Fecundity	No association between types of shift and duration of pregnancy attempt
[90]	Denmark	<25–35+	4763	809 (evening) 290 (night)	24,605	Fecundity	Fixed night and evenings had longer time to pregnancy
[11]	United States	38	673 (flight attendants)		91 (teachers)	Miscarriage	Increased risk for flight attendants who flew 15+ h during normal sleeping period from 10 PM to 8 AM
[13]	Denmark	30.5 ± 3.9	N/A	10,047	12,697	Miscarriage	Dose-dependent relationship between increased risk and successive night shifts, miscarriage rate for weeks 3–21 of pregnancy was lower for night shift group
[12]	United States	25–42	1766	680	5242	Miscarriage	Early miscarriage (<12 weeks): no increased risk for rotating shiftwork, 60% increase for nightshifts. Late miscarriage (12–20 weeks): 80% increase for nights only and 50% increase for rotating shiftwork, no nights
[91]	China	29.5	N/A	1860	N/A	Miscarriage	Working night shifts was independently associated with increased risk of miscarriage
[92]	Canada	N/A	90	75 (evening) 11 (night)	700	Miscarriage	Four times increased risk of pregnancy loss with evening work and more than twice as high for fixed nights
[93]	United States	25–42	1709	664	5109	Miscarriage	Failed pregnancies more likely from working night shift and long hours during first trimester
[94]	Denmark	<25–35+	6577	420	33,694	Miscarriage	Fixed night shifts associated with fetal loss
[95]	Sweden	<29–35+	1571	367	567	Miscarriage	Increased odds ratio for night shift and three-shift workers, increased odds ratio of late miscarriage for night shift workers
[96]	Sweden	N/A	44 (rotating) 285 (irregular)	11 (evening) 202 (night)	215	Miscarriage	Rotating shifts had slight increased risk of miscarriage
						Birthweight	Irregular work hours had significantly lower birth weights
[14]	United States	32.1 ± 4.5	N/A	N/A	131	Labor	Mean labor duration with: severe sleep disturbance, 26 h; normal sleep disturbance, 18.3 h; less than 6 h of sleep per night, 29 h; more than 7 h of sleep per night (normal), 17.7 h
						Delivery Type	Cesarean rate with: severe sleep disturbance, 39%; normal sleep disturbance, 10.3%; less than 6 h of sleep per night, 36.8%; more than 7 h of sleep per night (normal), 10.8%
[17]	Denmark	N/A	3197	400	32,465	Birthweight	Fixed nights: decrease 30 g in birthweight. Rotating shiftwork: no significant change
						Term Birth	No preterm association for rotating shiftwork, high risk of post-term for night workers
[97]	Canada	<20–29+	660 (changing shifts)	N/A	22,101	Birthweight	Increased risk for fatal ovarian cancer among rotating shift workers, but nightshift had no association
						Term Birth	Changing shift work less strongly associated with preterm birth
[16]	China	23.8 ± 2.5	127	N/A	38	Birthweight	All live birth: −79 g for rotating shiftwork. Production workers only: −109 g for rotating shiftwork
						Term Birth	All live birth incidence: 20% rotating shiftwork, 15.1% regular schedule. Production workers only incidence: 20.2% rotating shiftwork, 15.6% regular schedule
[98]	South Asia	26.4 ± 5.5	74		690	Birthweight	Shiftwork was a risk factor for small for gestational age less than 5^th^ centile
[99]	Ireland	16–44	133		529	Birthweight	Working 40+ hours per week and shiftwork was associated with a birthweight of 3000 g or less
[100]	Canada	<25–>35	N/A	177	900	Birthweight	Irregular shiftwork schedule increased risk for small for gestational age
[101]	Finland	N/A	368	N/A	1979	Birthweight	Increased number of small for gestational age for rotating shiftwork
[102]	United States	>16	N/A	166	1630	Term Birth	Night shifts have a 50% increased risk of preterm birth
[103]	Brazil	N/A	N/A	134	2164	Term Birth	No correlation with spontaneous preterm birth and nightshift
[104]	England	30.3 ± 3.8	N/A	131	1196	Term Birth	Preterm birth had little association with long working hours or shift work
[105]	Systematic review and meta-analysis					Term Birth	Night shifts or shifts not significantly associated with increased risk of preterm birth
[106]	Denmark	30.8	N/A	10,203	6298	Term Birth	No increased risk of preterm birth for night shift, risk not related to number, duration, or consecutive night shifts; women changing from night shifts in the first trimester to day work only in the second trimester had weak increased risk of preterm birth
[107]	Meta-analysis					Term Birth	Shiftwork and night shifts significantly associated with preterm birth
[108]	United States	35.5 ± 4.7	18,635		62,205	Menopause	Increased risk of early menopause. Women <45 years with exposure of 11–20+ years of rotating night shiftwork have 22% increased risk for early menopause
[109]	United States	30–55	46,801		31,761	Breast Cancer	Premenopausal: (years of shiftwork) 1–14 years had 23% increased risk 15+ years had 30% increased risk Postmenopausal: 30+ years had 45% increased risk
[110]	United States	30–55	31,442		22,045	Endometrial Cancer	20+ years of shiftwork had 47% increased risk, 2-fold increased risk for shiftwork women with a BMI of 30+
[111]	United States	35–74	N/A	831	2491	Invasive Ovarian Cancer	Any nightshift: 1.24-fold increased risk
						Borderline Ovarian Tumors	Any nightshift: 1.48-fold increased risk
[112]	United States	50.3	10,552	1754	141,637	Ovarian Cancer	Increased risk for fatal ovarian cancer among rotating shift workers, but nightshift had no association
[113]	United States	30–55 (NHS) and 25–42 (NHS11)	15,928		55,369	Ovarian Cancer	Rotating night shiftwork duration saw no association
[114]	United States	18–49	N/A	198	229	Endometriosis	Night shift work had 50% increased risk of endometriosis; risk doubled when women worked more than half of the shifts at night
[115]	United States	42.4 ± 3.7	419		244	Hormones	Recent night work (within last 2 weeks) had 56% decrease in urinary melatonin, long-term rotating shiftwork is associated with increased estradiol in postmenopausal women, no effect on progesterone levels
[116]	Spain	43.4 ± 12.15	63		73	Hormones	Rotating night shifts increased estradiol and progesterone levels, decreased testosterone levels, no significant difference in cortisol levels
[117]	Austria and Germany	24.2	10 subjects put under partial sleep deprivation			Hormones	LH, estriol, and thyroid-stimulating hormone levels increased, thyroid-stimulating hormone increasing significantly and remaining at high levels following sleep deprivation
[118]	United States	34.5	N/A	172	151	Hormones	Night shift workers: 6-sulfatoxymelatonin levels were lower and reproductive hormone levels were higher during daytime sleep and nighttime work, relative to nighttime sleep
[119]	Japan	22–39	9 (3 pregnant, 6 non-pregnant)		N/A	Hormones	Both: excretion profiles of urinary 6-sulfatoxymelatonin alter on night shift; urinary estriol level was not significantly affected by shift. Pregnant: urinary norepinephrine level during the night work was considerably higher; effect of night work on urinary estriol level of the pregnant women remained uncertain
[120]	United States	24	77		103	Hormones	Women having night/shift work had lower testosterone and increased LH relative to non-night/shift work women
[121]	Poland	40–60	N/A	263	269	Hormones	Frequency of night shift work did not determine hormone concentrations, total duration of night work tended to be positively associated with estradiol concentration, postmenopausal women with night work >15 years had increased estradiol levels

**Table 2:** Summary of outcomes in rodent light-shift models included in this review.

Publication	Rodent Species	Strain	Control Light Cycle	Experimental Light Cycle	Variables Measured	Results
[155]	Mouse	C57BL/6J	Stable LD12:12	Single 10 h phase shifts (advance/delay) and Chronic shifting (10 h advance for 3 days then 10 h delay for 4 days, for up to 9 months)	Estrous cycles, LH secretion profiles, natural fertility with 1 wk mating	Acute light shifts altered the LH surge and estrous cycling. Chronic shifting increased estrous acyclicity and pattern/amplitude of LH surge. Chronic shifting mice had reduced birth rate, number of pups/litter, and total number of pups
[76]	Mouse	Per2:Luciferase on C57 background	Food during dark phase	Normal; restricted food to light phase	Litter success rates, time to first litter, number of pups born and weaned, estrous cycling, mating, pregnancy maintenance	Light-restricted feeding reduced litters, reduced number of copulatory plugs, delayed time to first litter and did not change the number of pups born or weaned, estrous cycles, or pregnancy maintenance
[156]	Mouse	C57BL/6J Jms Slc and backcrossed with Cry1 or Cry2 KO		3 h delay of darkness onset for 2 days and then returned to the former phase of the LD cycles for 5 days	Estrous cycles, birth success	In Cry1 and Cry2 KO, light shifts altered estrous cycles and lowered pregnancy rates in older, but not young mice
[157]	Mouse	C57BL6	LD unchanged	12 h shift reversals for 36 days at the interval of 3, 6 or 12 days	Estrous cycles	Degree of circadian disruption was dependent on shift interval and estrous cycling was disrupted in all shift intervals
[158]	Mouse	Jcl:ICR	24 h day 12:12	22 (L11:D11) or 26 h day (L13: D13) light/ dark cycles for at least 2 weeks before mating and/or during pregnancy	Mating rate, pregnancy rate, resorption rates, number of pups	Mice on 22 h days or 26 h days had decreased mating rates, increased resorption and reduced pup rate with no change in pregnancy rate as compared to mice on 24 h days
[159]	Rats	Unknown	LD12:12	Long days (15 h light) reduced by 5 h either in AM or PM	Ovulation counts	Ovulation is adaptive to light. In the transition from long to shorter photoperiods, subtracting light from the AM (phase delay) results in delayed ovulation. Subtracting light from PM portion advanced ovulation
[160]	Rats	Albino Wistar	Stable LD12:12	Complete reversal of lights every 3–4 days	Pregnancy outcomes, growth and development; Offspring: metabolic, circadian, anxiety-like and behavioral despair	No differences in gestation length, litter size, survival to weaning, or birth weight. Offspring exhibited sex-specific metabolic effects
[161]	Rats	Albino Wistar	Stable LD12:12	Complete reversal of lights every 3–4 days	Pregnancy outcomes, metabolic function, circadian and metabolic gene expression	Light shifts disrupt weight gain across gestation (with similar food consumption), normal circadian melatonin, altered corticosterone, glucose, insulin, leptin, cholesterol, and free fatty acids. No change to glucose and insulin tolerance, Maternal liver clock gene expression (Bmal1 not rhythmic in shift, Per1 rhythmic but altered expression, Per2 not rhythmic, Rev-erba not rhythmic). Altered expression of metabolic genes in liver. Fetal gene expression also altered
[162]	Mouse	C57BL/6J	Stable LD 12:12	6 h advance or delay (4 times) every 5–6 days	Pregnancy outcomes, locomotor activity	Light advance group experienced the greatest reduction in the number of pregnancies caried to term. Light delays reduced the number of pregnancies caried to term as compared to no shifts

## Data Availability

Not applicable.
